# Sub-100-nm Nearly Monodisperse n-Paraffin/PMMA Phase Change Nanobeads

**DOI:** 10.3390/nano11010204

**Published:** 2021-01-14

**Authors:** Ho Young Woo, Da Won Lee, Tae Yeol Yoon, Jong Bae Kim, Ji-Yeon Chae, Taejong Paik

**Affiliations:** School of Integrative Engineering, Chung-Ang University, Seoul 06974, Korea; whymca0@gmail.com (H.Y.W.); leeda940811@gmail.com (D.W.L.); asd123490s@gmail.com (T.Y.Y.); jbkim0406@gmail.com (J.B.K.); jyeon10@cau.ac.kr (J.-Y.C.)

**Keywords:** phase change materials, colloid, nanoparticle, polymer, thermal energy storage

## Abstract

In this study, we demonstrate the colloidal synthesis of nearly monodisperse, sub-100-nm phase change material (PCM) nanobeads with an organic n-paraffin core and poly(methylmethacrylate) (PMMA) shell. PCM nanobeads are synthesized via emulsion polymerization using ammonium persulfate as an initiator and sodium dodecylbenzenesulfonate as a surfactant. The highly uniform n-paraffin/PMMA PCM nanobeads are sub-100 nm in size and exhibit superior colloidal stability. Furthermore, the n-paraffin/PMMA PCM nanobeads exhibit reversible phase transition behaviors during the n-paraffin melting and solidification processes. During the solidification process, multiple peaks with relatively reduced phase change temperatures are observed, which are related to the phase transition of n-paraffin in the confined structure of the PMMA nanobeads. The phase change temperatures are further tailored by changing the carbon length of n-paraffin while maintaining the size uniformity of the PCM nanobeads. Sub-100-nm-sized and nearly monodisperse PCM nanobeads can be potentially utilized in thermal energy storage and drug delivery because of their high colloidal stability and solution processability.

## 1. Introduction

Phase change materials (PCMs) have attracted significant attention for applications in thermal energy storage and management [1,2,3]. PCMs store thermal energy from the outer environment and release it when energy is needed during the phase change process [4]. The latent heat of organic or inorganic materials during phase change, including solid–solid, solid–liquid, and solid–gas or liquid–gas transitions, is commonly used for energy storage [5]. This phase change process is an isothermal process with small temperature variations and a large energy storage density, which allows PCMs to be utilized for a variety of applications such as for solar energy management [6,7], energy-saving buildings [8,9,10], thermal management for electronics [11,12,13,14,15], and textiles [16,17]. Among a variety of PCMs, organic PCMs including paraffin, fatty acids, alcohols, and polyglycols are particularly interesting owing to their large latent heat and small volume change during the phase transition [18]. Organic PCMs are relatively safe, non-corrosive, chemically stable, and have large phase change temperature variations depending on the type of organic PCM with low supercooling [19]. However, organic PCMs present problems pertaining to PCM leakage during the phase transition and low thermal conductivity, which limit their applications in thermal energy storage. As solid–liquid PCMs absorb energy during the solid-to-liquid melting process and release it during the solidification process, organic PCMs should be properly encapsulated to prevent the leakage of organic PCMs during the phase transition [20].

Shape-stabilized PCMs have been designed to enhance the stability of organic PCMs during the phase transition [21,22,23,24]. In this structure, PCMs are effectively secured within the supporting materials, which protect the organic PCMs from leakage during the phase transition. Various types of shape-stabilized PCMs have been reported, including core–shell structures [25], porous scaffolds [26,27], graphene-based networks [28,29], and polymeric structures [30,31]. Among these, polymethylmethacrylate (PMMA) has attracted much attention as a supporting material owing to its high thermal stability, high mechanical strength, nontoxicity, and low cost. Several studies on organic PCMs with PMMA as a supporting material have been reported [32,33,34]. However, the encapsulated PCMs are almost micron-sized or larger with a significant size variation. Compared to microencapsulated PCMs, nanoencapsulated PCMs have a larger surface area-to-volume ratio, leading to a higher heat transfer rate, which is expected to enhance the thermal conductivity of organic PCMs [35]. Furthermore, nanoencapsulated particles exhibit exceptional colloidal stability, indicating their potential for use as nanocarriers for thermosensitive drug delivery in biomedical applications [36,37,38,39,40]. In addition, the solution processability of colloidally stable PCMs offers low cost and facile integration of PCMs into stimuli-responsive nanocomposites [41,42,43,44].

In this study, we demonstrate the colloidal synthesis of sub-100 nm, nearly monodisperse n-paraffin/PMMA PCM nanobeads via emulsion polymerization. Emulsion polymerization is performed via free radical vinyl polymerization of the methylmethacrylate (MMA) monomer using ammonium persulfate as a radical initiator. Organic paraffin is encapsulated in the PMMA nanobeads, which effectively prevents the leakage of PCMs during the phase change process. The PCM nanobeads are sub-100 nm in size with a nearly monodisperse spherical shape and exhibit superior colloidal stability. The encapsulation of paraffin in the PMMA nanobeads and their phase transition behaviors were investigated using Fourier-transform infrared spectroscopy (FT-IR), thermogravimetric analysis (TGA), and differential scanning calorimetry (DSC). We further tailor the carbon length of n-paraffin used for the synthesis of the PCM nanobeads. The size and shape uniformity are not varied by changing the type of n-paraffin, while the phase transition temperatures of the PCM nanobeads increase with increasing carbon length.

## 2. Materials and Methods

### 2.1. Materials

All chemicals were used as purchased without further purification. n-Octadecane (99%), n-eicosane (99%), n-tetracosane (99%), MMA (99%), sodium dodecylbenzenesulfonate (SDBS, technical grade), and ammonium persulfate (98%) were purchased from Sigma-Aldrich (St. Louis, MO, USA). Deionized (DI) water was produced using a Millipore Milli-Q system.

### 2.2. Synthesis of Paraffin/PMMA Nanobeads

For the synthesis of the n-octadecane/PMMA nanobeads, 0.02 g of SDBS was dissolved in 42.5 mL of DI water and stirred under ambient atmosphere at 100 °C for 60 min. An oil phase solution was produced in which 1 g of n-octadecane was dissolved in 4 mL of MMA and stirred for 10 min at the same temperature. The solution was then cooled to 25 °C and ultrasonicated using a tip sonicator (Sonics and Materials VCX 750, Newtown, CT, USA) for 10 min. Next, the reaction temperature was increased to 80 °C under a nitrogen atmosphere. An initiator solution in which 0.04 g of ammonium persulfate was dissolved in 7.5 mL of DI water was added to the solution and the reaction temperature was maintained at 80 °C for 20 h. The resulting solution was washed with DI water and then redispersed in DI water. Two variants, n-eicosane/PMMA and n-tetracosane/PMMA nanobeads, were synthesized using n-eicosane and n-tetracosane instead of n-octadecane, respectively.

### 2.3. Characterization

Scanning electron microscopy (SEM) images were recorded using a Carl Zeiss SIGMA (Oberkochen, DE, Germany) operated at an accelerating voltage of 5 keV. Transmission electron microscopy (TEM) images were collected using a JEOL JEM-2100 (Akishima, Tokyo, Japan) instrument operating at 200 kV. Dynamic light scattering (DLS) was performed on an Otsuka Electronics DLS-7000 (Hirakata, Osaka, Japan) instrument. ζ-potential measurements were conducted using Otsuka Electronics ELS Z-1000. FT-IR was performed using a Bruker ALPHA II (Billerica, MA, USA). DSC was performed using a TA Instruments (New Castle, DE, USA) Discovery DSC system. DSC measurements were obtained at a heating or cooling rate of 10 °C/min under a nitrogen atmosphere. TGA was performed using a TA Instruments Discovery TGA instrument. TGA measurements were completed in the range of 25–600 °C at a heating rate of 10 °C/min under a nitrogen atmosphere.

## 3. Results

Phase change n-octadecane/PMMA nanobeads were synthesized via emulsion polymerization using SDBS as a surfactant. Emulsion polymerization and inverse emulsion polymerization are classified according to the formation conditions of the micelles. In emulsion polymerization, the hydrophobic group of the surfactant mixes with a hydrophobic reaction precursor, and the hydrophilic group of the surfactant is arranged in a surrounding hydrophilic reaction solvent to form micelles [45,46]. First, the water phase was formed by mixing the SDBS surfactant in DI water. Next, n-octadecane was mixed with MMA monomers to form the oil phase. After mixing the oil and water phases, ultrasonication was performed to form stable micelle structures.

Emulsion polymerization was performed via free radical vinyl polymerization of the MMA monomer using a persulfate radical initiator [45,47]. Ammonium persulfate was dissolved to form persulfate ions (S_2_O_8_^2−^), which subsequently dissociated into SO_4_^−•^ free radicals. These act as initiators for vinyl polymerization. A radical is unstable because it has an unpaired valence electron; therefore, SO_4_^−•^ free radicals prefer to collect an extra electron from the MMA monomers to become stable electronic structures. As the C=C bond of MMA monomers has a higher electron density than the C=O bond, SO_4_^−•^ free radicals react with the C=C bond in the MMA monomer and gain one electron to form a radical containing the MMA monomer. These unstable MMA radicals subsequently react with another MMA monomer to form a bond. As a result, PMMA polymers are generated via the subsequent polymerization of MMA monomers.

**Figure 1:**
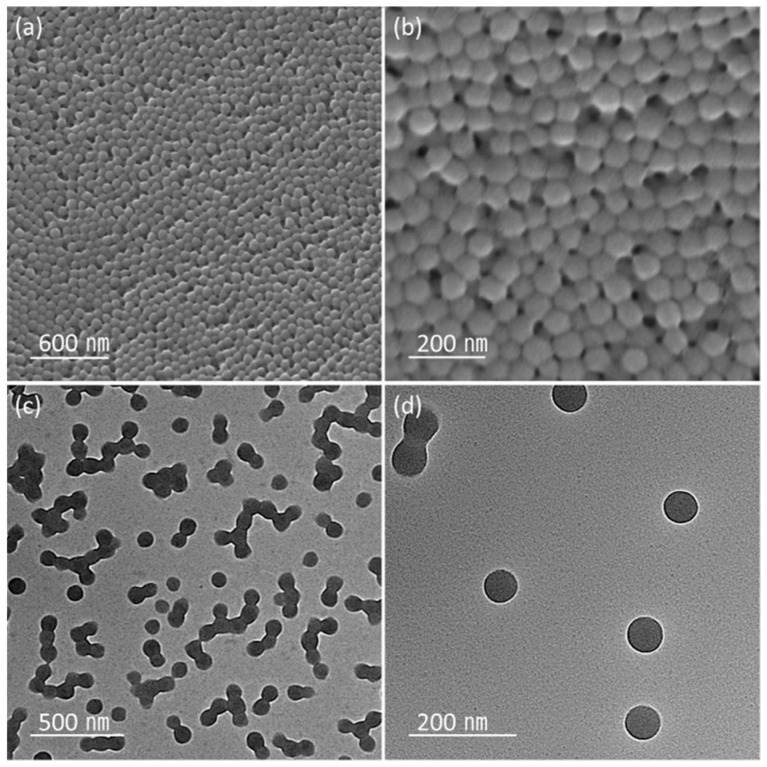
(**a**,**b**) SEM and (**c**,**d**) TEM images of n-octadecane/poly(methylmethacrylate) (PMMA) nanobeads.

Figure 1a–d shows the SEM and TEM images of the n-octadecane/PMMA PCM nanobeads. The low-magnification SEM image reveals nearly monodisperse spherical PCM nanobeads (Supplementary Materials Figure S1). Nanobeads with other shapes or organic impurities are not observed in the SEM images. Due to the high size uniformity, the PCM nanobeads self-assemble into close-packed superlattices, as displayed in Figure 1a,b. In addition, the n-octadecane/PMMA nanobeads present high colloidal stability in a DI water solution, as shown in Figure S1 (inset). Furthermore, the TEM images reveal the spherical morphologies of the n-octadecane/PMMA PCM nanobeads. The average particle diameter was measured to be 77.5 nm while the particle size distribution, calculated from a statistical size analysis of the TEM images, was determined to be 5.5 nm.

**Figure 2:**
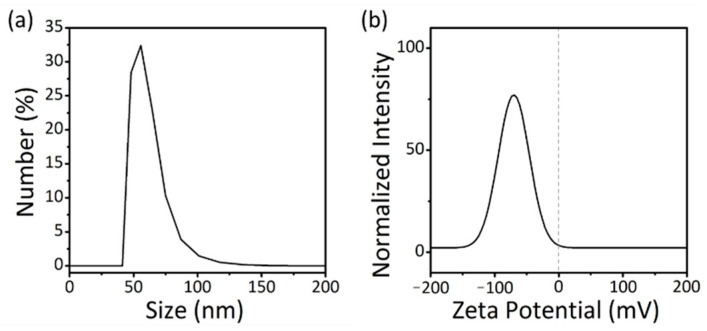
(**a**) Dynamic light scattering (DLS) and (**b**) ζ-potential measurements of n-octadecane/PMMA nanobeads.

To confirm the size and size uniformity of the PCM nanobeads, DLS measurements were conducted. The average particle diameter of the n-octadecane/PMMA nanobeads was confirmed to be 85.4 nm with a size deviation of 0.4 nm (Figure 2a). The average size measured by DLS was slightly larger than that calculated from TEM images, which is attributed to the fact that the size obtained by DLS includes the hydrodynamic radius of the water molecules around the n-octadecane/PMMA nanobeads; therefore, the overall size becomes slightly larger than that obtained from the TEM image analysis. The zeta potential was also obtained to investigate the surface charge of the n-octadecane/PMMA nanobeads (Figure 2b). This was measured as −73.97 mV, which can be attributed to the presence of SDBS on the surface of the n-octadecane/PMMA nanobeads, and the exposure of the negatively charged sulfone group of SDBS to the water phase. The zeta potential results indicate that electrostatic repulsion prevents agglomeration of the nanobeads. Therefore, n-octadecane/PMMA nanobeads exhibit high dispersibility and colloidal stability in DI water.

**Figure 3:**
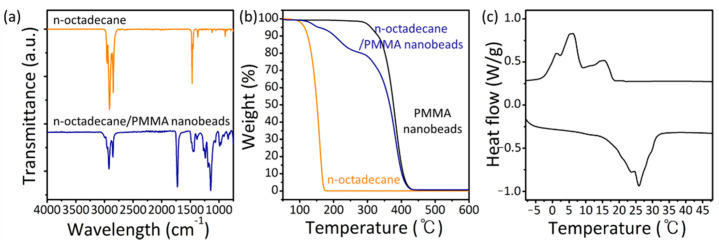
(**a**) FT-IR spectra of n-octadecane and n-octadecane/PMMA nanobeads. (**b**) Thermogravimetric analysis (TGA) of n-octadecane, PMMA nanobeads, and n-octadecane/PMMA nanobeads. (**c**) Differential scanning calorimetry (DSC) curves of n-octadecane/PMMA nanobeads.

To confirm the encapsulation of paraffin in the PMMA nanobeads, FT-IR measurements were conducted. Figure 3a displays the FT-IR spectra of the n-octadecane and n-octadecane/PMMA nanobeads. The n-octadecane/PMMA nanobeads exhibit characteristic vibration peaks near 1725 and 1144 cm^−1^ in the FT-IR spectrum, which are assigned to the C=O and C–O stretching vibrations of the ester group in the PMMA shell, respectively [48,49]. This reveals the presence of PMMA in the nanobeads. In addition, vibrational peaks at 2921 and 2851 cm^−1^ can be observed with the n-octadecane/PMMA nanobeads, which are attributed to symmetric and asymmetric C–H vibrations, respectively, indicating the presence of n-octadecane in the PCM nanobeads. We also synthesized PMMA nanobeads without n-octadecane (Figure S2). The SEM image shows that the size and morphology of the nanobeads are almost identical to those of the n-octadecane/PMMA nanobeads, with an average diameter of 69.1 ± 3.3 nm (Figure S2a). The peaks at 2993 and 2950 cm^−1^ are attributed to the stretching vibration of C–H, and characteristic peaks of the PMMA shell can be observed near 1725 and 1144 cm^−1^, which are attributed to the C=O and C–O stretching vibrations of the ester group (Figure S2b). Compared with the PMMA nanobeads, the PCM nanobeads show intense C–H stretching, which confirms the presence of n-octadecane in the PCM nanobeads.

The encapsulation of n-octadecane in the PMMA nanobeads was further confirmed by TGA. Figure 3b shows the thermogravimetric curves of n-octadecane, PMMA nanobeads, and n-octadecane/PMMA nanobeads. The weight loss of pure n-octadecane under heating can be observed at approximately 159.5 °C and the PMMA nanobeads began to decompose at approximately 382.1 °C. Both n-octadecane and PMMA nanobeads exhibit a single-step weight loss process, while the PCM nanobeads exhibit weight loss over a wider temperature range. When the temperature reached 140.1 °C, a weight loss of 5.2% occurred, which may be related to the presence of free n-octadecane. A further increase in the temperature to 216.1 °C induced a gradual weight loss of the PCM nanobeads by 14.0%, which is different from the behavior observed in the TGA curves obtained from n-octadecane and PMMA nanobeads. This is attributed to the decomposition of n-octadecane encapsulated in the PMMA nanobeads, resulting in an enhancement of the thermal stability of n-octadecane. The amount of encapsulated n-octadecane, calculated from the ratio of the relative weight loss of n-octadecane in the PCM nanobeads, was approximately 15.0%.

To investigate the phase change behavior, we conducted DSC measurements of the PCM nanobeads. Figure 3c and Figure S3a show the DSC measurement results of the n-octadecane/PMMA nanobeads and n-octadecane used as the PCM. Reversible melting and solidifying behaviors were observed in both samples. In the melting curve of the n-octadecane/PMMA nanobeads, a peak is located at 26.0 °C, which is slightly lower than the melting point of n-octadecane (Figure S3a). In the solidifying curve, peaks are located at 15.5, 6.2, and 1.3 °C, denoted α, β, and γ, respectively. Peaks α and β are attributed to heterogeneously nucleated liquid-rotator and rotator-crystal phase transitions, while peak γ is ascribed to a homogeneously nucleated liquid–crystal phase transition. The multiple peaks in the solidifying curve are related to the average diameter of the encapsulated PCMs. As the average diameter of encapsulated PCMs decreases, the number of nucleation sites decreases, and a liquid–crystal phase transition based on homogeneous nucleation occurs [50,51]. During the melting and solidifying process, the phase change temperature of the n-octadecane/PMMA nanobeads is lower than that of pure n-octadecane. The shift in the phase change temperature is related to the geometric confinement of PCMs in nanosized structures, and can be described by the Gibbs–Thomson effect [52,53]. According to the Gibbs–Thomson thermodynamic equation, the shift of the phase change temperature is inversely proportional to the pore size; thus, the nanosized structure produces a large shift in the phase change temperature.

The encapsulation efficiency (*E*) and encapsulation ratio (*R*) represent the phase change properties of the PCM nanobeads for thermal energy storage and can be calculated using the following equations [54]:

(1) $$E=\frac{\Delta H_{\mathrm{PCM}\;\mathrm{nanobeads}}}{\Delta H_{\mathrm{PCM}}}\times 100\%$$

(2) $$R=\frac{\Delta H_{m,\mathrm{PCM}\;\mathrm{nanobeads}}}{\Delta H_{m,\mathrm{PCM}}}\times 100\%$$

where Δ*H*_PCM nanobeads_ and Δ*H*_PCM_ are the sum of the melting and crystallization enthalpies of the PCM nanobeads and paraffin, respectively; Δ*H*_m,PCM nanobeads_, and Δ*H*_m,PCM_ are the melting enthalpies of the PCM nanobeads and paraffin, respectively. In the solidifying curve of n-octadecane (Figure S3a), the peaks are slightly twisted due to the supercooling of pure paraffin. Moreover, because of the supercooling, it is difficult to calculate the exact crystallization enthalpy of n-octadecane in the solidification curve. When calculating the n-octadecane content from the melting enthalpies of n-octadecane and the n-octadecane/PMMA nanobeads, the encapsulation ratio of the n-octadecane/PMMA nanobeads was calculated to be 14.3%, which is similar to the TGA results. Furthermore, we conducted thermal cycling tests to evaluate the thermal stability of PCM nanobeads. The DSC curves of the PCM nanobeads after 20 heating/cooling cycles still exhibit identical phase transition behaviors (Figure S4). These results indicate that the PMMA shell prevents leakage of the organic PCMs, and that the PCM nanobeads maintain stable phase change behavior over many phase transitions.

**Figure 4:**
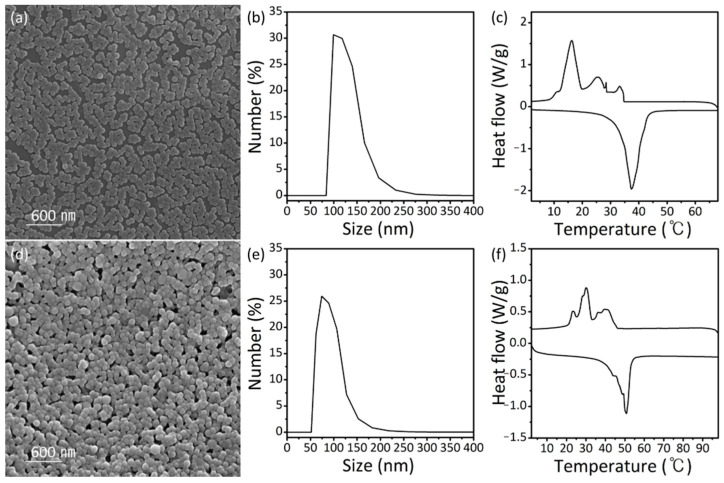
(**a**) SEM image, (**b**) DLS measurement, and (**c**) DSC curves of n-eicosane/PMMA nanobeads. (**d**) SEM image, (**e**) DLS measurement, and (**f**) DSC curves of n-tetracosane/PMMA nanobeads.

The phase change temperature of the PCM nanobeads can be further tailored by changing the hydrocarbon length of the n-paraffin. Figure 4a,d shows SEM images of the paraffin/PMMA PCM nanobeads synthesized with n-eicosane (C20) and n-tetracosane (C24), respectively. Spherical PCM nanobeads were synthesized with an average diameter of 168.7 nm using C20 paraffin and 149.7 nm using C24 paraffin, as characterized by DLS measurements (Figure 4b,e). The DSC measurements revealed an increase in the phase transition temperature with increasing hydrocarbon chain length (Figure 4c,f). In the melting curves of the n-eicosane/PMMA nanobeads and the n-tetracosane/PMMA nanobeads, peaks are located at 37.4 and 50.8 °C, respectively, which are consistent with the melting point of pure paraffin (Figure S3b,c). Similar to the n-octadecane/PMMA nanobeads, multiple peaks and a shift in the phase change temperature in the solidifying curves are observed, which are associated with the confined structure of the PCM nanobeads. The encapsulation ratios of the n-eicosane/PMMA nanobeads and n-tetracosane/PMMA nanobeads were calculated to be 34.9% and 17.4%, respectively.

## 4. Conclusions

We demonstrated the colloidal synthesis of nearly monodisperse, sub-100-nm n-paraffin/PMMA PCM nanobeads via emulsion polymerization. Organic paraffin is encapsulated within PMMA nanobeads, which effectively prevents the leakage of PCMs during the phase change process. When n-octadecane is used as an organic PCM, the PCM nanobeads are sub-100 nm in size with a nearly monodisperse spherical shape and exhibit superior colloidal stability. We further tailored the carbon length of n-paraffin used for the synthesis of the PCM nanobeads. The size and shape uniformity do not vary by changing the type of n-paraffin, whereas the phase change temperature of the PCM nanobeads increases with increasing carbon length of n-paraffin. Sub-100-nm-sized and nearly monodisperse PCM nanobeads can be potentially utilized for thermal energy storage and drug delivery because of their high colloidal stability and solution processability.

## Data Availability

The data presented in this study are available on request from the corresponding author.

## Supplementary Materials

The following are available online at https://www.mdpi.com/2079-4991/11/1/204/s1, Figure S1: Low-magnification SEM image of n-octadecane/PMMA nanobeads. The inset image displays a photograph of the n-octadecane/PMMA nanobeads in a DI water solution, Figure S2: (a) SEM image and (b) FT-IR spectra of PMMA nanobeads, Figure S3: DSC curves of (a) n-octadecane, (b) n-eicosane, and (c) n-tetracosane.
