# Supplementary material for: Sub-100-nm Nearly Monodisperse n-Paraffin/PMMA Phase Change Nanobeads
Source: Nanomaterials (Basel). 2021 Jan 14;11(1):204. doi: 10.3390/nano11010204 (PMC7830838; doi:10.3390/nano11010204)
Supplement: Supplementary file 1 [file nanomaterials-11-00204-s001.pdf]

nanomaterials

Supplementary Materials

Sub-100-nm Nearly Monodisperse n-Paraffin/PMMA Phase Change Nanobeads

Ho Young Woo, Da Won Lee, Tae Yeol Yoon, Jong Bae Kim, Ji-Yeon Chae, and Taejong Paik*

School of Integrative Engineering, Chung-Ang University, Seoul 06974, Korea; whymca0@gmail.com (H.Y.W); leeda940811@gmail.com (D.W.L.); asd123490s@gmail.com (T.Y.Y.); jbkim0406@gmail.com (J.B.K.); jyeon10@cau.ac.kr (J.-Y.C.)

*Correspondence: paiktae@cau.ac.kr (T.P.)

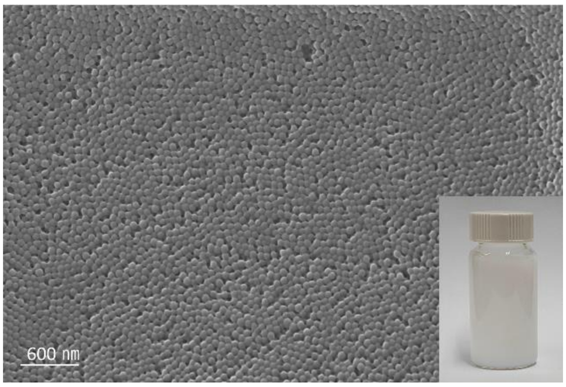

Figure S1. Low-magnification SEM image of n-octadecane/PMMA nanobeads. Inset image displays a photograph of the n-octadecane/PMMA nanobeads in a DI water solution.

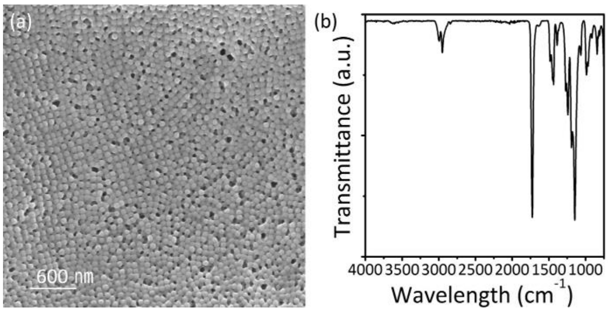

Figure S2. (**a**) SEM image and (**b**) FT-IR spectra of PMMA nanobeads. Emulsion polymerization conducted without n-octadecane.

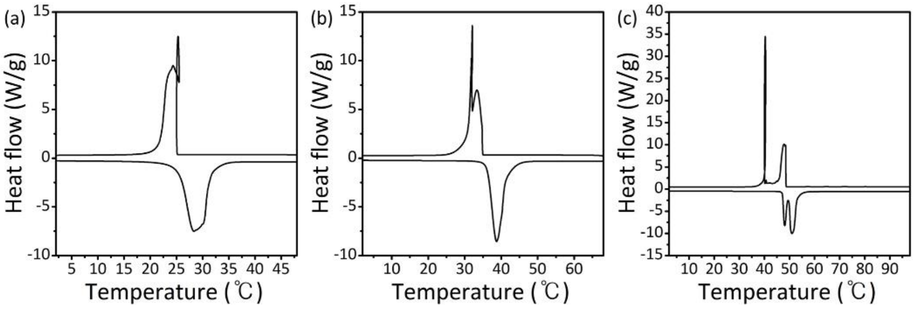

Figure S3. DSC curves of (**a**) n-octadecane, (**b**) n-eicosane, and (**c**) n-tetracosane.

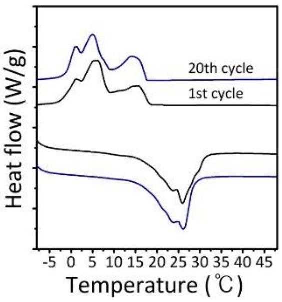

Figure S4. DSC curves for 20 phase change cycles of n-octadecane/PMMA nanobeads.
